# Gene targeting and transgene stacking using intra genomic homologous recombination in plants

**DOI:** 10.1186/s13007-016-0111-0

**Published:** 2016-02-01

**Authors:** Sandeep Kumar, Pierluigi Barone, Michelle Smith

**Affiliations:** Dow AgroSciences LLC, 9330 Zionsville Road, Indianapolis, IN 46286 USA

**Keywords:** Gene targeting, Plant transformation, Transgene stacking, Designed nuclease, Intra genomic homologous recombination, Somatic recombination

## Abstract

Modern agriculture has created a demand for plant biotechnology products that provide durable resistance to insect pests, tolerance of herbicide applications for weed control, and agronomic traits tailored for specific geographies. These transgenic trait products require a modular and sequential multigene stacking platform that is supported by precise genome engineering technology. Designed nucleases have emerged as potent tools for creating targeted DNA double strand breaks (DSBs). Exogenously supplied donor DNA can repair the targeted DSB by a process known as gene targeting (GT), resulting in a desired modification of the target genome. The potential of GT technology has not been fully realized for trait deployment in agriculture, mainly because of inefficient transformation and plant regeneration systems in a majority of crop plants and genotypes. This challenge of transgene stacking in plants could be overcome by Intra-Genomic Homologous Recombination (IGHR) that converts independently segregating unlinked donor and target transgenic loci into a genetically linked molecular stack. The method requires stable integration of the donor DNA into the plant genome followed by intra-genomic mobilization. IGHR complements conventional breeding with genetic transformation and designed nucleases to provide a flexible transgene stacking and trait deployment platform.

## Background

The Green Revolution in the 1960s combined advances in breeding and agricultural practice, and provided food security to millions of people [1]. Given an increasing global population, there is a projected need to increase world food production by 40 % in the next 20 years [2]. In addition to a growing population, climate change, degrading natural resources and changing food preferences have raised food and nutritional security to the level of the biggest challenge of the twenty-first century [3].

Genetically modified (GM) trait technology in the mid-1990s made a major impact in meeting the world food demand and there has been a rapid adoption of the technology. These first generation trait products involved simple herbicide and insect traits that required introduction of a single gene. Control of the broad range of insect pests and weeds desired today requires multiple insect and herbicide tolerance genes [4]. In addition, modern genomics and gene networking tools have revealed that many agronomic traits depend on different genes and complex interactions of proteins reacting to various external stimuli [1]. The next generation trait products, therefore, require integration of multiple transgenes and would also benefit from a flexible and modular trait stacking platform that would accommodate development of increasingly complex future products. Conventional breeding has been successfully employed for trait stacking, but this method requires substantial time and resources for sorting and deregulation of multiple unlinked transgenes [4, 5], and a limited number of independent loci can practically be stacked.

Designed nucleases have become a powerful gene targeting (GT) tool to create targeted DNA double strand breaks (DSBs) at specified genomic locations, which stimulate the cell’s DNA repair machinery leading to integration of exogenously supplied transgenes into a specified genomic site. While designed nuclease-mediated targeted mutagenesis is becoming routine in plants [6–9], site-directed transgene integration remains elusive, mainly due to low transformation and regeneration efficiencies in the majority of plant species and genotypes. A GT method that requires minimal transformation effort would be very attractive to address this challenge. This review focuses on conventional Intra-chromosomal somatic homologous recombination work in plants and its recent application using designed nucleases that can provide solutions to some of the challenges associated with the deployment of GT technology for transgene stacking in crop plants.

## Gene targeting: a byproduct of genomic double-strand break

DSBs can arise spontaneously, may be induced by ionizing radiation and chemicals, or recently by designed nucleases (For review, see references [10–15]). Genomic DSBs could be negatively mutagenic or lethal to cells if not repaired efficiently. In plants, DSBs are repaired by homologous recombination (HR) or non-homologous end joining (NHEJ). HR and NHEJ mechanisms are conserved in eukaryotes; however, the efficiency of these pathways differs not only between species but also between cell types [16]. HR is a precise DSB repair pathway that requires sequences homologous (almost identical) to those flanking the DSB site [12, 13]. HR is the predominant DNA recombination pathway during meiosis in higher eukaryotes including plants [17]. NHEJ mainly involves ligation to unrelated sequences or to sequences with micro-homologies, resulting mostly in non-precise repair with small insertions or deletions at the DSB site. NHEJ is the primary DNA repair pathway in the somatic cells, while HR mainly occurs during S and G2 phases of the cell cycle [18].

Targeted DSB-induced NHEJ has been previously described for mutagenesis, deletions or imprecise insertions [6–9, 13, 19, 20]. In contrast, HR, a more precise mode of DNA repair, is preferred for GT [12, 13]. Gene targeting through HR requires simultaneous introduction of the nuclease to create targeted DSB at desired genomic location, and donor DNA containing flanking homologies, acting as a template for repair of the DSB [21].

## Gene targeting challenges in plants

**Fig. 1:**
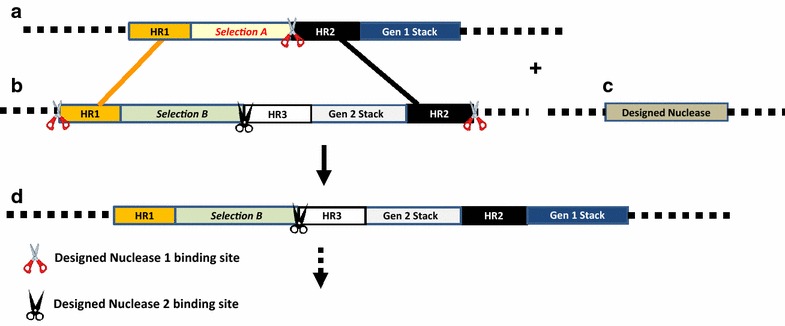
Basic design of constructs used for gene targeting via intra genomic homologous recombination. **a** Target construct contains a generation 1 (Gen 1) stack and a selection marker A flanked by unique homology sequences (HR1 and 2). A designed nuclease 1 binding site is inserted between selection marker A and HR2 sequence. **b** The donor construct contains selection marker B, downstream HR3 sequence, generation 2 (Gen 2) stack, and HR1 and 2 homology sequences matching the target. A designed nuclease 2 binding site is inserted between the selection marker and HR3 sequence for future targeting. The donor is flanked by a designed nuclease 1 binding site on each end. **c** The designed nuclease construct contains designed nuclease 1 coding sequence driven by appropriate promoter. **d** Target locus containing functional selection marker B gene and generation 2 (Gen 2) stack precisely inserted after gene targeting

Targeted DSBs stimulate the cell’s DNA repair machinery making the DSB site accessible to a donor transgene for site-specific integration. The DSBs however do not preclude the ectopic integration of a donor transgene elsewhere in the genome. In addition, the GT process requires efficient delivery of the donor molecule to the DSB site and the ability to regenerate whole plants from the cells with a precisely repaired targeted genomic site. Random integration of the donor transgene and an inefficient transformation method for the donor delivery therefore are two major challenges for the routine deployment of GT technology in crop plants. Positive selection for GT such that precise insertion of the donor complements the non-functional selectable marker in the target locus has been used to avoid random integration of the donor [22, 23, 24] genes in the target locus. A positive–negative selection approach has also been used very successfully for GT in rice [25, 26]. A sequential GT method providing flexibility of incremental modifications of the target locus with new trait genes was recently developed [27]. That method exploited positive GT selection using an intron sequence homology between the donor and target that allowed sequential swapping of selection markers, providing a multi-generational GT method (Fig. 1) for trait product deployment [28].

The accessibility of the donor transgene to the DSB site is another key bottleneck for efficient GT process. Donor DNA is exogenously supplied either via direct DNA delivery [29], mostly using the microparticle bombardment, or via indirect DNA delivery, mainly mediated by *Agrobacterium* [30]. The production of a transgenic plant is the result of a sequence of events: a) transfer of exogenous DNA into the plant cell nucleus, b) integration of the foreign DNA in a transcriptionally active region of the host genome, and c) regeneration into a fully developed plant, either via organogenesis or somatic embryogenesis, of the original cell where the transgene integrated. Regardless of the gene transfer method used (direct or indirect) cell competence for foreign DNA integration and regeneration varies with cell type and developmental stage making the recovery of transgenic events a challenging task in most crop plants.

The nuclear targeting of the exogenous DNA is hindered by physical (e.g. cell wall), cellular (e.g. proteases, nucleases) and biological barriers (e.g. plant defense) and our understanding of how to best overcome these barriers is still limited [31, 32]. Actively dividing cells are the most amenable targets for DNA insertion and it has been shown that higher transformation is obtained in cells with nuclei at the S and G2 phases [33, 34] where chromatin remodeling takes place. A localized and temporary decondensation of the chromatin is believed to be necessary for efficient transgene integration in gene-rich euchromatic regions [35, 36].

Plant biology parameters associated with species, genotype, and explant type play an important role in the efficiency of transformation and regeneration. In rice, for example, between the two subspecies indica and japonica, indica is generally more recalcitrant to tissue culture and transformation [37]. Similarly for maize transformation and regeneration, the most responsive type of explant is the immature embryo where scutellum cells are induced to proliferate and undergo somatic embryogenesis [38], but this process is highly genotype-dependent and still limited mainly to crosses and derivatives of the maize inbred lines A188 [37]. Also in soybean, the ability to regenerate transgenic plants has been limited to a few soybean model genotypes (Jack and Williams 82) with some successful examples of competence for somatic embryogenesis transferred and combined in other cultivars via introgression [39].

## Gene targeting via intra genomic homologous recombination

The challenges of inefficient transformation systems in crop plants could be overcome by intra-genomic homologous recombination (IGHR), which utilizes a cell’s recombinational machinery to replicate and supply donor DNA for IGHR mediated insertion of a donor within the target site. Intra-chromosomal HR in somatic cells of the whole plant was reported more than two decades ago (Reviewed in [40, 41, 42]). Two overlapping, non-functional pieces of a chimeric *beta-glucuronidase* (*uidA*) gene were used as recombination substrates, which upon HR led to a restoration of the functional *uidA* gene that was detected by histochemical staining of the encoded functional *uidA* protein. HR was reported in different organs and tissues during different stages of the plant development, including meristematic recombination events that revealed cell lineage patterns. The system was later used to demonstrate that an induced DSB in the target site resulted in twofold increase the HR frequency [22, 43]. The germline in plants is formed during later developmental stages, and any HR occurring during the life cycle of the plant could be germinally transmitted to the next generation. The demonstration of HR between linked overlapping DNA sequences within somatic cells of the whole plant was an important milestone in the GT field. The work paved the way for HR between unlinked DNA sequences in the genome of somatic cells, and regeneration of whole plants from these cells (see below).

The next significant development in the field was the application of designed nucleases for excision of the stably integrated transgene. In tobacco, a transgenic line containing a single copy of the *codA* gene flanked by cleavage sites specific to I-*Sce*I nuclease was created. After induction of DSBs by transient expression of I-*Sce*I, the *codA* gene was successfully removed from the calli, and plants lacking the *codA* gene were regenerated [44]. Similarly, tobacco plants containing a stably integrated *uidA* gene cassette flanked by designed nuclease sites were crossed with plants expressing the corresponding nuclease. The complete deletion of a 4.3 kb sequence comprising the *uidA* gene cassette was obtained in F_1_ progenies [45]. These reports were later followed by deletions of large endogenous genomic sequences in different plant species using designed nucleases [46–48].

Researchers in mammalian GT field were first to exploit cells’ recombination machinery to catalyze HR between a target locus and an in vivo-liberated donor [49]. In this system, the donor transgene is first inserted stably into the genome. The randomly inserted donor molecule is later released intragenomically within the genome of intact tissue. The IGHR based method was demonstrated using a site-specific recombinase (FLP) and a site-specific endonuclease (I-*Sce*I) for the modification of the *yellow* locus in the *Drosophila* genome [49–51]. The method has been successfully applied for the modification of more than 20 loci in *Drosophila* [52].

A similar IGHR approach was also proposed for plant GT [53]; the first proof-of-principle in plants came several years later in *Arabidopsis* [54] using a single site-specific endonuclease (I-*Sce*I). The GT system was designed using a non-functional truncated *uidA* target transgene containing cleavage sites for I-*Sce*I nuclease, a donor transgene containing a complementary *uidA* GT cassette flanked by I-*Sce*I sites, and a transgene containing a I-*Sce*I expressing cassette that upon expression would generate in vivo release of linear donor after I-*Sce*I expression. Single copy target and donor lines were crossed and lines homozygous for both the transgenes were obtained. The homozygous target/donor lines were then crossed with an I-*Sce*I line and F_1_ progenies were screened for IGHR-mediated GT using *uidA* histochemical staining. Some F_1_ progenies revealed chimeric blue spots indicating GT in somatic cells during the plant development. The F_1_ lines were self-pollinated and F_2_ progenies were scored for the blue seedlings indicating germinal transmittance of GT. Targeted events were obtained up to one per 100 seeds. A similar approach was later attempted with some success in a native genomic target site in *Arabidopsis* using the CRISPR/Cas system [55].

After initial work on IGHR-mediated GT in a model system, the method was successfully demonstrated in maize by somatic ectopic recombination and tissue culture selection [56]. Similar to a previous effort in *Arabidopsis*, the target construct contained a non-functional partial *neomycin phophotransferase II* (*nptII*) gene and a cleavage site for I-*Sce*I nuclease. The donor construct contained dexamethasone-inducible I-*Sce*I, and an excisable *nptII* sequence complementing the partial sequence at the target locus such that GT would constitute the functional *nptII* gene. The target and donor plants were crossed and F_1_ progenies were selfed. No fully kanamycin-resistant plants were obtained from the dexamethasone-induced F_2_ progeny for target and donor. However kanamycin-resistant leaf sectors were observed indicating IGHR occurred in some somatic cells during plant development. The embryos isolated from immature kernels of F_2_ plants were subjected to callus induction on medium with and without dexamethasone. Kanamycin resistant GT events were recovered and the repair of the *nptII* gene was confirmed by molecular analyses. GT frequencies ranging from 0.13 to 0.55 % (per immature embryo treated) were obtained. The authors also made an interesting observation of GT at a cleaved target locus without excision of the donor molecule.

The demonstration of GT via IGHR in *Arabidopsis* and maize has created potential for the application of GT technology in transformation-inefficient crop plant species. Unlike direct transformation methods that limit donor molecules to a small number of treated cells, IGHR utilizes the plant system to replicate donor DNA in every cell throughout the life cycle. The extra-chromosomal donor molecule could be liberated and used by the target site as a template in plant tissues or stages that favor HR over NHEJ. The previous GT approaches relied on efficient transformation systems to produce a large number of events to obtain a few targeted plants. Since most economically important crop plants remain recalcitrant to transformation, GT technology has so far been practical in only a small number of crop plants. Additionally, IGHR releases only one to two copies of the donor, leading to high quality targeted events, in contrast to previous GT methods that require additional segregation work to remove randomly integrated undesired truncated donor molecules.

**Fig. 2:**
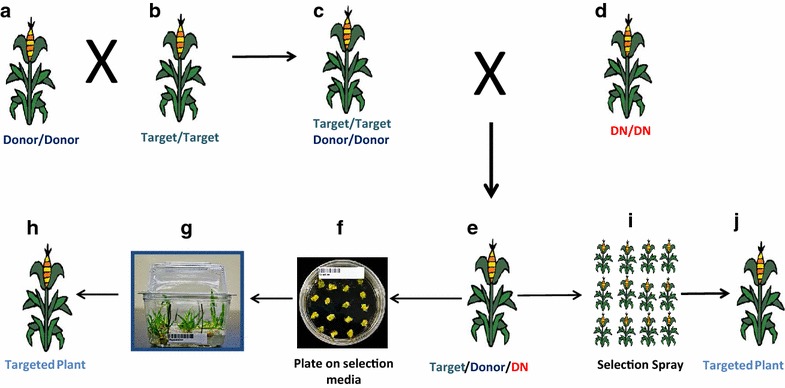
Crossing and targeted plant production strategy in maize using intra-genomic homologous recombination. Plants homozygous to donor (**a**) and target (**b**) are crossed and self-pollinated to obtain progenies that are homozygous to target and donor loci (**c**). The homozygous target donor plants are crossed with plants containing designed nuclease (DN) transgene (**d**) to obtain F_1_ progenies transgenic to target, donor and DN (**e**). The F_1_ immature embryos are treated on appropriate selection media (**f**) and targeted plants are regenerated on selection (**g** and **h**). Alternatively, the F_1_ plants could be selection sprayed (**i**) to obtain targeted plants (**j**)

The IGHR method reviewed here generates tremendous opportunity for biotechnological application of GT in commercial transgenic trait deployment. This approach when combined with a sequential GT method (Fig. 1) [27] would provide the modular and flexible transgenic trait stacking platform (Fig. 2) currently needed for complex product needs in the agriculture industry. The strategy provides flexibility to stably integrate 1st generation or geography-specific traits in the target plant, while new traits are placed in the donor plant. Donor and target plants are crossed to create a breeding stack, which is then crossed with appropriate designed nuclease expressing plants. The F_1_ progenies are then subjected to tissue culture selection and targeted plant regeneration. Multiple donor lines containing different traits could strategically be made to keep modularity required for creating on-demand stacked transgenic traits. The additional tissue culture selection step restricts the use of this method to crop plants that are amenable to tissue culture techniques. Precise tissue-specific expression of designed nuclease in reproductive cells [57–59] can circumvent the need for a tissue culture regeneration process, providing broader application of this approach across different crops.

## Conclusions

Future biotech crops are projected to require multiple transgenes to confer resistance to a broad spectrum of insect pests and provide herbicide tolerance with different modes of action. Insects and weeds will eventually develop resistance, new target pests will emerge and new traits will inevitably be needed and desired, so designing those future products to be further modified and developing capabilities to accomplish the modifications are wise investments. It is clear that producing and modifying transgenic events through GT has many advantages over random integration, and technology continues to develop to make GT increasingly efficient and flexible. Intra genomic homologous recombination using designed nucleases has good potential to overcome limitations in plant transformation and breeding to achieve targeted and highly complex stacked trait crops.

## Abbreviations

DSBs: double strand breaks
GT: gene targeting
IGHR: intra-genomic homologous recombination
HR: homologous recombination
NHEJ: non-homologous end joining
*uidA*: *beta*-*glucuronidase*
*nptII*: *neomycin**phophotransferase**II*
